# Supplementary material for: Content-rich biological network constructed by mining PubMed abstracts
Source: BMC Bioinformatics. 2004 Oct 8;5:147. doi: 10.1186/1471-2105-5-147 (PMC528731; doi:10.1186/1471-2105-5-147)
Supplement: Additional File 2 — The original results of the above study (non-essential files are deleted to keep the file size under the limit set by BMC bioinformatics). [file 1471-2105-5-147-S2.bz2 › chilibotAdditionalFile2/dip30/92ID8950973E399/html/GRB2_CBL.html]

 


 **GRB2** and **CBL** 
  
Found 100 abstracts in PubMed, retrieved 30.  
 

 What does Google say? 
 PDF only 
| .edu only 

---

**Interactive relationship** (e.g. stimulation, inhibition, etc)

**Neutral relationship**- In vitro binding studies using GST fusion proteins revealed that interactions of  **Grb2**  Shc and CrkL  **Cbl**  were mediated through each SH2 domain in tyrosine phosphorylation dependent manner.  Ref: 11792427 Mol Immunol, 2002
- SETA is a multifunctional adapter protein with three SH3 domains that binds  **Grb2** ,  **Cbl** , and the novel SB1 proteins.  Ref: 11152963 Cell Signal, 2000
- Evidence that SETA binds to the CD2 protein, the proto oncogene c  **Cbl**   [ **CBL** ] , and the signal transduction molecule  **Grb2** , and can dimerize via its C terminal coiled coil CC domain is also presented.  Ref: 11152963 Cell Signal, 2000
- Interaction of some Ruk isoforms with several signalling proteins, including the p85 regulatory subunit of the Class IA PI 3 kinase, c  **Cbl**  and  **Grb2** , has been demonstrated.  Ref: 12242006 Gene, 2002
- Localization of  **Cbl**  yellow fluorescent protein to these endocytic organelles was dependent on a proline rich domain of c  **Cbl**  that interacts with  **Grb2**  as shown by fluorescence resonance energy transfer microscopy.  Ref: 12839496 Traffic, 2003
- L selectin tyrosine phosphorylates  **cbl**  and induces association of tyrosine phosphorylated  **cbl**  with crkl and  **grb2** .  Ref: 11263968 Biochem Biophys Res Commun, 2001
- These data suggest that the binding of  **Cbl**  to epidermal growth factor receptor through  **Grb2**  is necessary and sufficient for  **Cbl**  function during clathrin mediated endocytosis.  Ref: 12839496 Traffic, 2003
- Immunodepletion studies reveal that  **Grb2**  associated tyrosine phosphorylated p120 kDa protein is  **Cbl** .  Ref: 11399323 Mol Immunol, 2000
- **Grb2**  and Shc are also degraded in an EGF induced  **Cbl**  b dependent fashion.  Ref: 11375397 J Biol Chem, 2001
- In vitro binding studies using GST fusion proteins demonstrate that  **Cbl**  constitutively associates with the SH3 domains of  **Grb2** , with a preference for the amino terminal domain.  Ref: 11399323 Mol Immunol, 2000
- Thus, in addition to its well characterized role in mediating positive signals,  **Grb2**  can terminate signal transduction by accelerating c  **Cbl**  dependent sorting of active tyrosine kinases to destruction.  Ref: 11823423 EMBO J, 2002
- Although Ron and c  **Cbl**  are found also in physical complexes that include  **Grb2** , these associations are insufficient for productive ubiquitylation of Ron.  Ref: 12802274 Oncogene, 2003
- **Cbl**   **Grb2**  and  **Cbl**  CrkL interactions have been implicated in T cell and B cell receptor, and cytokine receptor signaling.  Ref: 11399323 Mol Immunol, 2000
- Here we analyzed tyrosine phosphorylation and interactions of  **Cbl**  with adapter proteins,  **Grb2**  and CrkL, in NK3.3 cells.  Ref: 11399323 Mol Immunol, 2000
- Differential interaction of  **Cbl**  with  **Grb2**  and CrkL in CD2 mediated NK cell activation.  Ref: 11399323 Mol Immunol, 2000
- The data are consistent with the model whereby  **Grb2**  participates in EGFR internalization through the recruitment of  **Cbl**  to the receptor, thus allowing proper ubiquitylation of EGFR and or associated proteins at the plasma membrane.  Ref: 12631709 Mol Biol Cell, 2003
- Ligand induced desensitization of the epidermal growth factor receptor EGFR is controlled by c  **Cbl** , a ubiquitin ligase that binds multiple signaling proteins, including the  **Grb2**  adaptor.  Ref: 11823423 EMBO J, 2002
- Phosphorylation of LAT creates binding sites for the Src homology 2 SH2 domains of other proteins, including PLC gamma1,  **Grb2** , Gads, Grap, 3BP2, and Shb, and indirectly binds SOS, c  **Cbl** , Vav, SLP 76, and Itk.  Ref: 11752630 Sci STKE, 2000
- Overexpression of c  **Cbl**  mutants that are capable of  **Grb2**  binding but defective in linker RING finger domain function severely inhibited epidermal growth factor receptor internalization.  Ref: 12839496 Traffic, 2003
- **Grb2**  SH3 domains formed an association with the proline rich sequences of Sos and  **Cbl**  in both resting and activated platelets, since the peptidimer abolished these associations.  Ref: 11964172 Biochem J, 2002
- CD2 crosslinking markedly induced tyrosine phosphorylation of  **Cbl**  associated with  **Grb2**  or CrkL, Shc and LAT, compared with IL 2 stimulation.  Ref: 11792427 Mol Immunol, 2002
- Here, we demonstrate that  **Grb2**  bound to tyrosine phosphorylated FRS2 alpha forms a ternary complex with  **Cbl**  by means of its Src homology 3 domains resulting in the ubiquitination of fibroblast growth factor FGF receptor and FRS2 alpha in response to FGF stimulation.  Ref: 11997436 Proc Natl Acad Sci U S A, 2002

**Non-interactive relationship** (e.g. studied together, co-existance, homology, etc.)

- The signaling proteins, Vav,  **Grb2** ,  **Cbl**  and Gab2, and the endocytic proteins, AP2 and clathrin, all map to the primary domains, while the p85 regulatory subunit of phosphatidylinositol 3 PI 3 kinase maps to both domains.  Ref: 12217393 Mol Immunol, 2002
- Although the precise roles of interactions of  **Cbl**  with  **Grb2**  and CrkL in NK cell activation remains to be elucidated, their tyrosine phosphorylation, in addition to the multiple protein interactions described here, strongly suggest that interactions of  **Cbl**  with  **Grb2**  and CrkL may play pivotal roles in CD2 mediated NK cell activation.  Ref: 11399323 Mol Immunol, 2000
- Co immunoprecipitations showed EGFR in complex with both Shc,  **Grb2**  and  **Cbl** .  Ref: 11370743 Eur J Cell Biol, 2001
- However, costimulation of IL 2 with CD2 crosslinking remarkably enhanced associations of  **Grb2**  Shc and CrkL  **Cbl** , compared to IL 2 stimulation or CD2 crosslinking alone.  Ref: 11792427 Mol Immunol, 2002
- Utilizing unique phospho specific antibodies generated against various tyrosines of c Met, we show that Y1003 binding site for c  **Cbl**  and a negative regulatory site, Y1313 binding site for PI3K, Y1230 Y1234 Y1235 autophosphorylation site, Y1349 binding site for  **Grb2** , Y1365 important in cell morphogenesis are phosphorylated in response to HGF 40 ng ml, 7.5 min in H69 cells.  Ref: 12611639 J Cell Mol Med< MedlineTA>J Cell Mol Med, 2002
- It was found in complexes with many signaling proteins, including phosphoinositol PtdIns 3 kinase EC 2.7.1.137,  **Cbl** ,  **GRB2** , p130Cas and Crk.  Ref: 12135478 Eur J Biochem, 2002
- EphA activation also induced  **Cbl**  association with Crk L and Crk II adapters, but not the related  **Grb2**  protein.  Ref: 12794130 J Immunol, 2003
- Furthermore, the EGFR mutant lacking  **Grb2**  binding sites did not efficiently recruit c  **Cbl**  and was not polyubiquitinated.  Ref: 12631709 Mol Biol Cell, 2003
- We show that a substantial fraction of tyrosine phosphorylated epidermal growth factor receptor and Shc,  **Grb2**  and  **Cbl**  after internalization relocates from early endosomes to compartments which are negative for the early endosomes, recycling vesicle markers EEA1 and transferrin in EGF stimulated cells.  Ref: 11370743 Eur J Cell Biol, 2001
- FRS2 alpha attenuates FGF receptor signaling by  **Grb2**  mediated recruitment of the ubiquitin ligase  **Cbl** .  Ref: 11997436 Proc Natl Acad Sci U S A, 2002
- About 50% of the T cells from young mice that formed conjugates with peptide pulsed APC were found to form complexes, at the site of binding to the APC, containing CD3epsilon, linker for activation of T cells LAT, and Zap 70 in a central area and c  **Cbl** , p95 vav, Grb 2  [ **GRB2** ] , PLC gamma, Fyn, and Lck distributed more uniformly across the interface area.  Ref: 11207267 J Immunol, 2001
- The transmembrane adapter LAT coprecipitates with SLP 76 and PLCgamma2, as well as with a number of other adapter proteins, some of which have not been previously described in platelets, including  **Cbl** ,  **Grb2** , Gads, and SKAP HOM.  Ref: 10942756 J Biol Chem, 2000
- The underlying mechanism seems to involve recruitment of a  **Grb2**  c  **Cbl**  complex to  **Grb2**  specific docking sites of EGFR, and concurrent acceleration of receptor ubiquitylation and desensitization.  Ref: 11823423 EMBO J, 2002
- In contrast to constitutive  **Cbl**  association with  **Grb2** , tyrosine phosphorylated  **Cbl**  interacts with CrkL via its SH2 domain only after CD2 stimulation.  Ref: 11399323 Mol Immunol, 2000
- Our results shed light on the mechanism of receptor desensitization mediated by c  **Cbl**  and its binding partner  **Grb2** .Oncogene 2003 2 669 3679.  Ref: 12802274 Oncogene, 2003
- The PER mutant with all five autophosphorylation sites mutated to phenylalanine 5YF was equivalently capable of interacting with several important signaling molecules, including Shc,  **Grb2** , Gab1, phospholipase Cgamma, and  **Cbl** .  Ref: 12615082 Int J Dev Neurosci, 2003
- There is an activation induced association of tyrosine phosphorylated  **Cbl**  with  **Grb2**  and CrkL, respectively, but not CrkII.  Ref: 11263968 Biochem Biophys Res Commun, 2001
- NTAL becomes rapidly tyrosine phosphorylated upon cross linking of the B cell receptor BCR or of high affinity Fcgamma and Fc varepsilon receptors of myeloid cells and then associates with the cytoplasmic signaling molecules  **Grb2** , Sos1, Gab1, and c  **Cbl** .  Ref: 12486104 J Exp Med, 2002
